# Establishment of a Steatosis Model in LMH Cells, Chicken Embryo Hepatocytes, and Liver Tissues Based on a Mixture of Sodium Oleate and Palmitic Acid

**DOI:** 10.3390/ani14152173

**Published:** 2024-07-26

**Authors:** Wuchao Zhuang, Ziwei Chen, Xin Shu, Jilong Zhang, Runbang Zhu, Manman Shen, Jianfei Chen, Xiaotong Zheng

**Affiliations:** 1 Jiangsu Key Laboratory of Sericultural and Animal Biotechnology, School of Biotechnology, Jiangsu University of Science and Technology, Zhenjiang 212100, China; zhuangwuchao123@163.com (W.Z.); chenziwei20222022@163.com (Z.C.); shu18574525727@163.com (X.S.); zhangjilong0930@163.com (J.Z.); r13zhu@163.com (R.Z.); mms@just.edu.cn (M.S.); 2 Key Laboratory of Silkworm and Mulberry Genetic Improvement, Ministry of Agriculture and Rural Affairs, Sericultural Scientific Research Center, Chinese Academy of Agricultural Sciences, Zhenjiang 212100, China

**Keywords:** fatty acid, RNA-seq, LMH, chicken embryo liver, lipid metabolism

## Abstract

**Simple Summary:**

Establishing a cellular steatosis model is crucial for studying liver lipid deposition in poultry. This study used leghorn male hepatoma (LMH) cells to investigate the effects of oleic acid (OA), sodium oleate (SO), palmitic acid (PA), sodium palmitate (SP), and their pairwise combinations on steatosis development. Subsequently, RNA-seq was employed to comprehensively analyze alterations in gene expression within cells under various steatosis-inducing conditions. In primary chicken embryonic liver cells and liver tissue, the optimal conditions for inducing steatosis identified in this study can also induce steatosis effectively. Overall, this study found that a combination of SO and PA efficiently induces steatosis in various chicken liver cell types and chicken embryonic liver tissues.

**Abstract:**

Research on hepatic steatosis in animal husbandry has been a prominent area of study. Developing an appropriate in vitro cellular steatosis model is crucial for comprehensively investigating the mechanisms involved in liver lipid deposition in poultry and for identifying potential interventions to address abnormalities in lipid metabolism. The research on the methods of in vitro liver steatosis in chickens, particularly the effects of different fat mixtures, is still lacking. In this study, LMH cells were utilized to investigate the effects of OA, SO, PA, SP, and their pairwise combinations on steatosis development, with the aim of identifying the optimal conditions for inducing steatosis. Analysis of triglyceride (TG) content in LMH cells revealed that OA and SP had limited efficacy in increasing TG content, while a combination of SO and PA in a 1:2 ratio exhibited the highest TG content. Moreover, Oil Red O staining results in LMH cells demonstrated that the combination treatment had a more pronounced induction effect compared to 0.375 mM SO. Additionally, RNA-seq analysis showed that 0.375 mM SO significantly influenced the expression of genes associated with fatty acid metabolism compared to the control group, whereas the combination of SO and PA led to an enrichment of key GO terms associated with programmed cell death. These findings suggest that varying conditions of cellular steatosis could lead to distinct disruptions in gene expression. The optimal conditions for inducing steatosis in LMH cells were also tested on chicken embryonic liver cells and embryos. TG detection and Oil Red O staining assays showed that the combination of SO and PA successfully induced steatosis. However, the gene expression pattern differed from that of LMH cells. This study lays the foundations for further investigations into avian hepatic steatosis.

## 1. Introduction

The excessive accumulation of triglycerides (TG) in liver cells, referred to as hepatic steatosis [1,2], can lead to the development of fatty liver syndrome (FLS) and fatty liver hemorrhage syndrome (FLHS) in chickens if left untreated. FLHS is particularly prevalent in cage farming conditions, significantly impacting the breeding of commercial laying hens and broilers [3,4]. Non-alcoholic fatty liver disease (NAFLD) in humans poses a serious health threat [5]. Given the great similarities between chicken FLHS and NAFLD [6], investigating the mechanisms underlying FLHS production may provide valuable insights for the analysis of NAFLD. Recently, numerous studies have focused on the lipid metabolism of the liver in poultry, with a specific emphasis on investigating the underlying molecular mechanisms and exploring potential additives to alleviate FLS [7,8,9,10,11]. Addressing excessive liver fat deposition is crucial for enhancing economic outcomes in the poultry industry and promoting poultry health.

In order to investigate the development of fatty liver and identify potential alleviating additives, researchers typically establish in vivo or in vitro models of steatosis. In vitro models offer advantages including expedited experimentation and enhanced stability in comparison to in vivo models, rendering them more conducive for investigative purposes. Numerous investigations have been conducted on liver cell steatosis models in both human and mammalian subjects, with well-established methods for model development. Human-derived liver cancer (HepG2) cell lines exhibit rapid proliferation rates and epithelioid morphology, enabling the execution of various differentiated liver functions. Consequently, researchers frequently employ HepG2 cell steatosis models to elucidate the mechanisms underlying liver cell steatosis and the potential effects of specific substances on NAFLD [12]. Palmitic acid (PA) is a prevalent saturated free fatty acid found in both the human body and food sources, frequently utilized in research on metabolic liver diseases to replicate liver cell damage resulting from lipotoxicity [13]. Hepatic steatosis is more commonly associated with unsaturated fatty acids; oleic acid (OA), the most abundant monounsaturated fatty acid in the body, is often employed as a model for liver steatosis in vitro [13]. The combination of PA, OA, and sodium oleate (SO) has been shown to induce lipid accumulation in HepG2 cells, establishing an in vitro model for NAFLD [14,15]. In addition to HepG2 cells, L02 (human normal hepatocytes) cells, AML12 (mouse liver cell line) cells, and THLE-2 (transformed human liver epithelial-2) cells are also good liver cell models. Wang et al. simulated hepatic steatosis by culturing L02 cells with OA [16]; Xiang et al. used PA/OA (0.375/0.75 mM) to intervene in AML12 cells and establish a model of cell steatosis [17]. Cheng et al. induced steatosis in THLE-2 cells using OA and PA [18].

Several research studies exist on the development of cellular steatosis models in poultry. Zhang et al. utilized a 10% fat emulsion to culture primary liver cells in chickens, resulting in the successful establishment of a fatty liver degeneration model in broiler chicken embryos [19]. Song et al. demonstrated that inducing leghorn male hepatoma (LMH) with 0.375 mM SO for 12 h can effectively induce steatosis [20]. This is also currently the optimal condition for single fatty acid-induced steatosis in LMH cells. Subsequent studies have indicated that conditions leading to fatty degeneration in mammalian cell lines can also induce steatosis in primary chicken liver cells [9,21]. Nevertheless, there is a lack of comprehensive investigation into the impact of various lipid solutions and combinations of fatty acids on the steatosis of poultry liver cells. The objective of this study is to determine the optimal conditions for inducing steatosis in LMH cells by utilizing various lipid solutions and to examine the resulting gene expression profiles using RNA-seq analysis. Subsequently, the optimal conditions identified for inducing steatosis in LMH cells were applied to primary chicken liver cells and chicken embryo liver tissue to evaluate their impact on steatosis and gene expression. This research aims to provide a favorable method for inducing steatosis in LMH cells, chicken embryo liver cells, and liver tissue, thereby providing a basis for further investigation into the pathogenesis and therapeutic strategies for poultry fatty liver.

## 2. Materials and Methods

### 2.1. Animals and Sample Collection

Animal protocols were approved by the Institutional Animal Care and Use Committee (IACUC) of Jiangsu University of Science and Technology (G2022SJ13, Zhenjiang, China). Animal care and handling were performed according to the IACUC guidelines.

The embryonic eggs of the Hongyao chicken, an indigenous breed in China, were incubated under controlled conditions with a temperature of 37.8 °C and a relative humidity of 60%, following established hatchery procedures. After 14 days of incubation, a portion of eggs were utilized for the isolation of primary liver cells. Subsequently, at 17 days of incubation, the embryonic eggs were divided into 3 groups of 5 each, which included a negative control (NC) group, a group treated with 0.375 mmol of SO, and a group treated with 0.45 mmol SO/PA mixture (the ratio is 1:2) and incubated through in ovo feeding technology. Different lipid culture media were injected into chicken embryos through in ovo feeding technology; the injected eggs were cultured for an additional 2 days before being sampled for analysis. Chicken embryo liver tissue was harvested and divided into three portions, which were then frozen in enzyme-free EP tubes and stored at −80 °C for the determination of TG content, Oil Red O staining, and RNA extraction.

### 2.2. Cell Isolation, Culture, and Treatment

Chicken primary embryo hepatocytes were isolated from 14-day incubated eggs using a method outlined by Zhang et al. [22]. The primary liver cells were cultured in dulbecco’s modified eagle medium (DMEM) (Gibco, Shanghai, China) supplemented with 1% penicillin/streptomycin at 37 °C/5% CO_2_ in an incubator. LMH cells were cultured in DMEM mixture F-12 (DMEM/F12) (Gibco) plus 10% superfine fetal bovine serum (BioChannel Biotechnology Co., Ltd., Nanjing, China), 1% penicillin/streptomycin at 37 °C/5% CO_2_ in an incubator. The cell culture plate was coated with 0.1% gelatin for 1 h, washed twice with PBS, and then cells were seeded in a 24-well plate. Before treatment, freshly stock solutions with a concentration of 50 mM were prepared to induce steatosis. When preparing OA and SO stock solutions, a 0.5% BSA solution was used as the solvent, while a mixed solution of NaOH and isopropanol was used as the solvent for preparing PA and SP stock solutions. To prepare the latter, the process involved adding 0.1 M NaOH solution and heating in a 70 °C water bath until the solid dissolved. The solution was then allowed to cool before adding an equal amount of isopropanol to the NaOH. The mixture was heated at 70 °C until completely dissolved, followed by filtration of the resulting fluid for subsequent use. At approximately 80% confluence, for use in the treatment of cells, the stock solution was diluted into the required concentration with a complete cell culture medium. For in ovo feeding assays, the stock solution was diluted with 100 μL/egg PBS before injection.

### 2.3. TG Content Detection

TG content in LMH cells, primary hepatocytes, and liver tissues was quantified following the manufacturer’s guidelines provided with the commercial kits from Nanjing Jiancheng Bioengineering Institute (Nanjing, China). The BCA protein quantification kit from Vazyme was used to determine protein concentration for the normalization of TG content. Absorbance readings were measured using a BioTek Epoch 2 microplate auto-reader from BioTek (Winooski, VT, USA).

### 2.4. Oil Red O Staining

LMH cells and primary chicken hepatocytes were stained using the Modified Oil Red O Staining Kit from Senbeijia Biotechnology (Nanjing, China), following the manufacturer’s instructions. Frozen sections of chicken embryo liver were dehydrated with sucrose, embedded in OCT, and then sectioned and stained with Oil Red O. Tissue morphology and structure were observed using pathological image scanners OLYMPUS VS200 from OLYMPUS (Tokyo, Japan), with a 20× objective.

### 2.5. RNA Isolation, RNA-Seq Sequencing, and Read Mapping

Total RNA was extracted using Trizol reagent (Accurate Biology, Changsha, China), and its concentration and quality were assessed using a Nanophotometer N60 Touch (IMPLEN, Munich, Germany). RNA integrity was evaluated using the RNA Nano 6000 Assay Kit on an Agilent 2100 Bioanalyzer system (Agilent Technologies, Santa Clara, CA, USA). Subsequently, mRNA sequence libraries were prepared with the NEBNext Ultra RNA Library Prep Kit for Illumina (New England Biolabs, Inc., Ipswich, MA, USA), and paired-end reads were sequenced on an Illumina Novaseq 6000 platform by Gene Denovo Biotechnology Co. (Guangzhou, China). Raw reads underwent processing with fastp (v0.18.0) to remove adapters, poly-N regions, and low-qualit reads, resulting in a set of clean reads. Quality metrics, including Q20, Q30, and GC content, were computed for the clean data. The clean reads were then aligned to the chicken genome assembly (GRCg7b) utilizing Hisat2 (v2.1.0). A reference genome index was generated with Hisat2 (v2.1.0), and then clean paired-end reads were aligned to this index. Mapped reads from each sample were then assembled using StringTie (v1.3.4) in a reference-based manner. Subsequently, for each transcription region, a TPM (transcripts per kilobase of exon model per million mapped reads) value was determined to quantify its expression abundance and variations, employing RSEM software (v1.2.19). Differential expression analysis between the two groups was utilizing the DESeq2 R package (v1.20.0).

### 2.6. Reverse Transcription Quantitative Real-Time Polymerase Chain Reaction

cDNA was synthesized from 1 μg of the extracted total RNA using the Evo M-MLV RT Mix Kit with gDNA Clean (Accurate Biology). Reverse transcription quantitative real-time polymerase chain reaction (RT-qPCR) was performed using a Bio-Rad Light Cycler 96 Real-Time PCR system with Magic SYBR mixture (CoWin Biosciences, Taizhou, China). The specific RT-qPCR system and amplification program were the same as in previous studies [23]. *TBP* and *WAC* were selected as internal reference genes in this study. All RT-qPCR gene-specific primers (Table S1) were designed using Primer Premier 5.0 software.

### 2.7. Bioinformatics and Statistical Analysis

Principal component analysis (PCA) was conducted using the R package gmodels (http://www.r-project.org/) accessed on 18 March 2024. Gene ontology (GO) and kyoto encyclopedia of genes and genomes (KEGG) enrichment analysis of differentially expressed genes (DEGs) was performed with the clusterProfiler R package (version 3.8.1), which included correction for gene length bias. Protein–protein interaction analysis was carried out using the STRING v10 online software (http://cn.string-db.org) accessed on 9 May 2024. The network file was visualized using Cytoscape software (version 3.7.1) to illustrate core and hub gene biological interactions. RT-qPCR data were collected and analyzed using Bio-Rad CFX Manage (v3.1). The CT values of the genes were exported to Microsoft Excel (v2016), and the relative gene expression levels were calculated using the 2^−ΔΔCT^ method. Statistical analysis was conducted using ANOVA with Tukey’s multiple comparison test, with significance determined at *p* < 0.05, *p* < 0.01, or *p* < 0.001.

## 3. Results

### 3.1. Identification of the Optimal Conditions for STEATOSIS in LMH Cells

**Figure 1:**
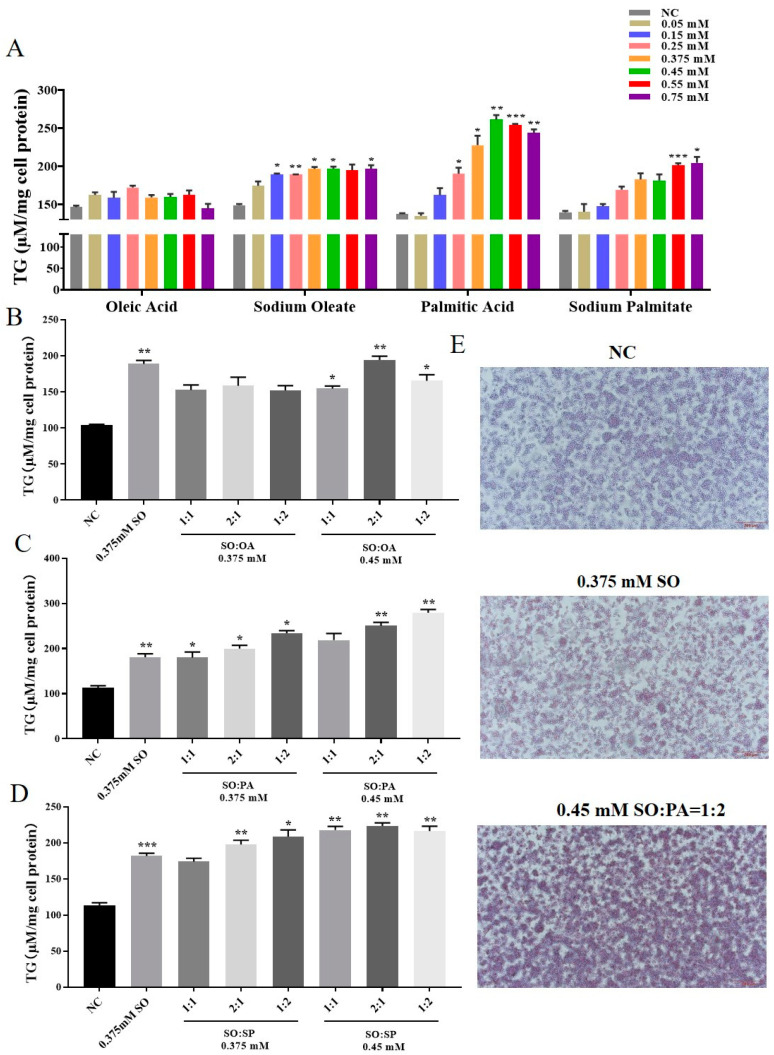
The effect of different lipidation conditions on lipidation of LMH cells. (**A**) The effects of different concentrations of OA, PA, and their sodium salts on the TG content of LMH cells. (**B**–**D**) represent the detection of TG content in LMH cells after 12 h of combined cultivation of SO with OA, PA, and SP, respectively. (**E**) Staining results of Oil Red O in LMH cells under different conditions. Error bars indicate the SE (*n* = 3). Abbreviations: NC means negative control; SO means sodium oleate; PA means palmitic acid; SP means sodium palmitate. Significant differences relative to the NC group were denoted by asterisks (* *p* < 0.05, ** *p* < 0.01, *** *p* < 0.001).

In order to determine the optimal conditions for steatosis in LMH cells, we exposed the cells to 4 different fatty solutions: OA, SO, PA, and SP. Concentration gradients ranging from 0 to 0.75 mM were applied to the cells. The TG assay kit was utilized to measure the TG content within the cells, as illustrated in Figure 1A. The impact of OA on inducing cell steatosis was found to be inconsistent, whereas SO demonstrated a more consistent effect. PA was determined to have the most significant impact on inducing cellular steatosis. Moreover, at the concentrations of 0.375 mM and 0.45 mM for SO and PA steatosis, the cellular TG content was significantly higher than in other groups. Then, we explore optimal conditions for cell steatosis using a combination of SO and three other solutions (OA, PA, and SP) at concentrations of 0.375 mM and 0.45 mM in ratios of 1:1, 2:1, and 1:2. The 2 control groups consisted of 0.5% BSA and SO at 0.375 mM. Based on the findings of the TG analysis, it was observed that the combination of SO and OA exhibited the most pronounced steatosis effect. Conversely, the combinations of SO with PA and OA demonstrated better steatosis effects compared to a concentration of 0.375 mM of SO alone. However, it was noted that the TG content of the SO and PA combination was lower than that of the 0.375 mM SO group. The Oil Red O staining results also showed the steatosis effect of SO/PA (0.15/0.3 mM) was better than that of SO at 0.375 mM (Figure 1E). Therefore, it can be inferred that the optimal combination for achieving the steatosis effect was SO and PA, with the most favorable outcome observed at a ratio of 1:2.

### 3.2. RNA-Seq Analysis of LMH Cells under Different Steatosis Conditions

**Figure 2:**
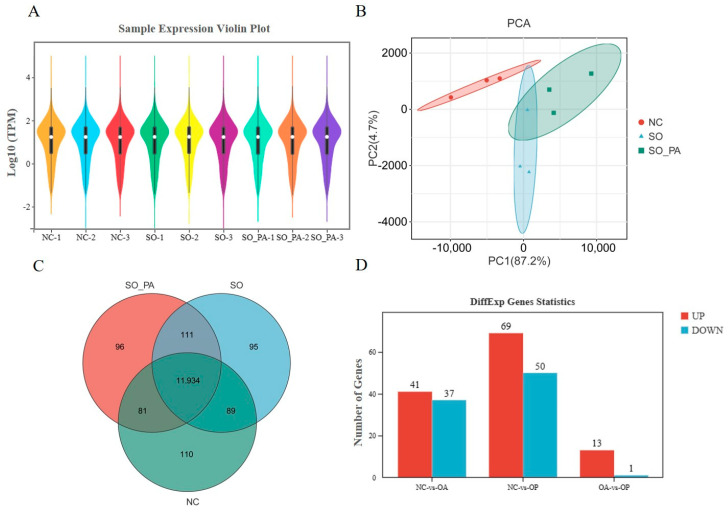
Parameter statistics of gene expression levels in the RNA-seq analysis. (**A**) Violin plot of the gene expression of all samples. (**B**) PCA analysis. (**C**) Venn diagram of the expressed genes. (**D**) Statistics of differentially expressed genes between different groups. Abbreviations: NC means negative control; SO means sodium oleate; PA means sodium palmitate.

**Figure 3:**
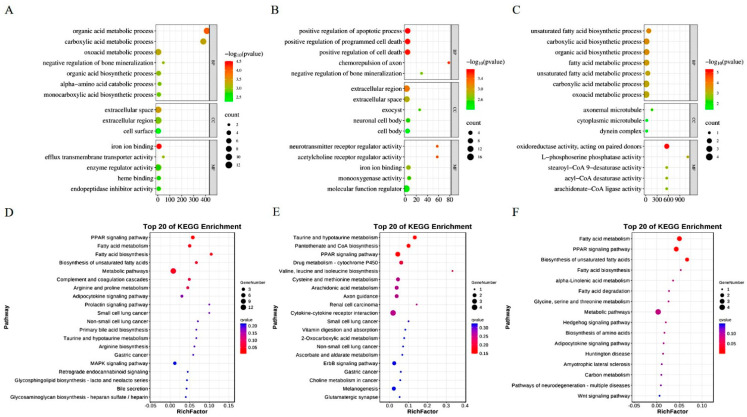
Functional enrichment analysis of differentially expressed genes identified in the RNA-seq. (**A**–**C**) represent GO enrichment analysis of DEGs of different groups, respectively: NC vs. SO, NC vs. SO + PA, and SO vs. SO + PA. (**D**–**F**) represent KEGG enrichment analysis of DEGs of different groups, respectively: NC vs. SO, NC vs. SO + PA, and SO vs. SO + PA. Abbreviations: NC represents negative control; SO means 0.375 mM sodium oleate; SO + PA represents the mixture of sodium oleate and palmitic acid.

**Figure 4:**
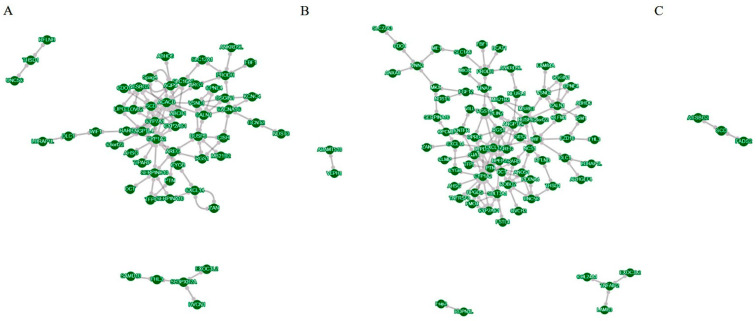
Protein–protein association networks of the differentially expressed genes: (**A**–**C**) represent enrichment results of different groups, respectively: NC vs. SO, NC vs. SO + PA, and SO vs. SO + PA. Abbreviations: NC represents negative control; SO means 0.375 mM sodium oleate; SO + PA represents the mixture of sodium oleate and palmitic acid.

To examine the molecular biology mechanism underlying the induction of steatosis in LMH cells within the optimal conditions established in this study, three treatment groups were established: a 0.375 mM SO treatment group, a SO and PA mixed solution treatment group, and an NC group. Following a 12 h treatment period, cellular RNA was extracted for sequencing. Assessment of the raw data quality and read mapping results indicated that the RNA-seq sequencing exhibited high quality and was suitable for subsequent analyses (Tables S2 and S3). The gene expression profiles of the samples exhibited similarity (Figure 2A). PCA analysis indicated effective separation of the three treatment groups along the first principal component (Figure 2B). The RNA-seq analysis screened 11,934 expressed genes in all groups (Figure 2C). Through the identification of DEGs with a |log_2_ foldchange| > 1 and a corrected *p* value < 0.05, it was observed that there were 78, 139, and 14 DEGs in the NC vs. SO, NC vs. SO + PA, and NC vs. SO + PA groups, respectively (Figure 2D). Functional enrichment analysis was performed on DEGs, and the results showed that fatty acid metabolism-related GO terms and pathways were significantly enriched in the NC vs. SO groups (Figure 3A,D), while cell death-related GO terms and KEGG pathways were significantly enriched in the NC vs. SO + PA groups (Figure 3B,E). Furthermore, to SO vs. SO + PA, the fatty acid metabolism processes were the most significantly enriched terms (Figure 3C,F). The protein–protein interaction network analysis results further indicated the differential core and hub gene biological interactions between different groups (Figure 4).

### 3.3. Quantitative Validation of Differentially Expressed Genes Identified through RNA-Seq

**Figure 5:**
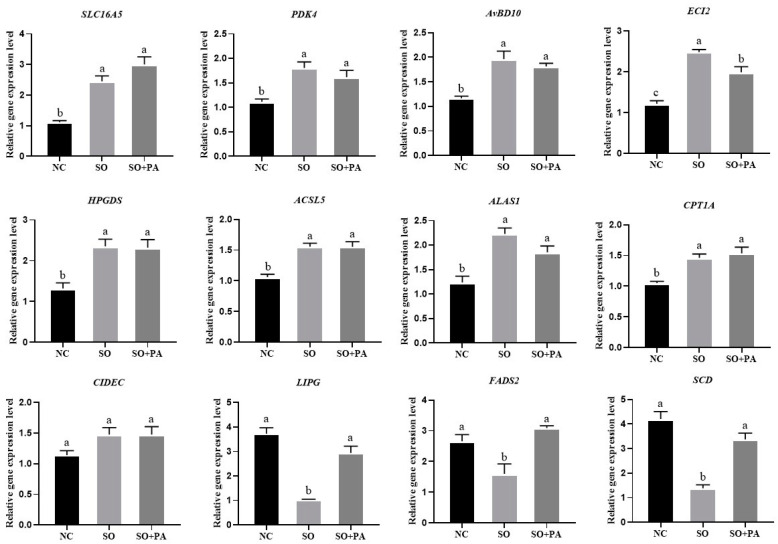
Gene expression levels of differentially expressed genes in LMH cells under different induction conditions. Abbreviations: NC represents negative control; SO means 0.375 mM sodium oleate; SO + PA represents the mixture of sodium oleate and palmitic acid. Different lowercase letters indicate significant differences (*p* < 0.05).

In order to further confirm the accuracy of RNA-seq, we validated 11 DEGs related to lipid metabolism and 1 antimicrobial peptide gene *AvBD10* through RT-qPCR. As depicted in Figure 5, of the 12 selected DEGs, *SLC16A5*, *PDK4*, *AvBD10*, *ECI2*, *HPGDS*, *ACSL5*, *ALAS1*, and *CPT1A* genes showed significantly higher expression in the treatment group compared to the NC group. *CIDEC* expression was higher in the treatment group but not significantly expressed. *LIPG* and *SCD* genes had significantly lower expression in the SO treatment group compared to the NC group and SO + PA group. The validation results of RT-qPCR indicated that the RNA-seq results are reliable.

### 3.4. The Application of Optimal Conditions for Steatosis in Chicken Primary Embryonic Liver Cells and Embryonic Liver Tissues

**Figure 6:**
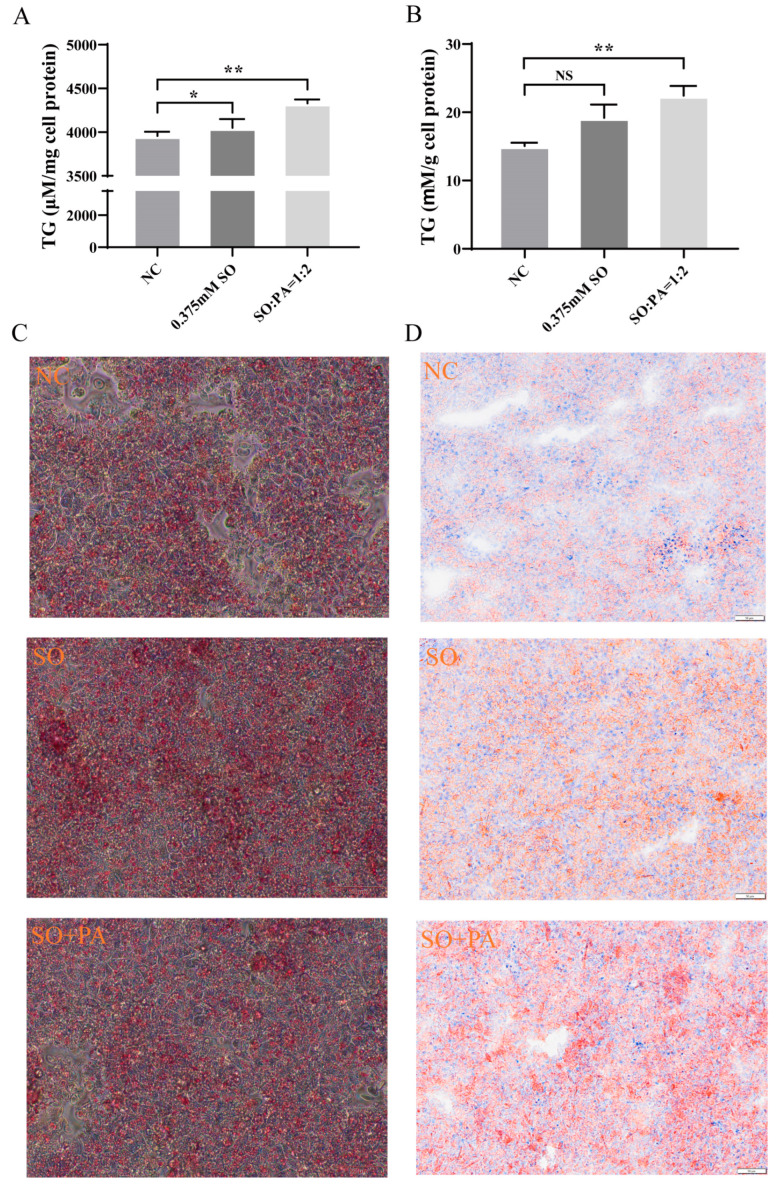
The effect of optimal steatosis method on the lipid content of primary chicken embryo liver cells and chicken embryo liver tissue. (**A**) Detection of TG content in primary cells of chicken embryo liver. (**B**) Detection of TG content in chicken embryo liver tissue. (**C**,**D**) The Oil Red O staining results of primary chicken embryo liver cells and liver tissue, respectively. Abbreviations: NC represents negative control; SO means 0.375 mM sodium oleate; SO + PA represents the mixture of sodium oleate and palmitic acid. Significant differences were denoted by asterisks (* *p* < 0.05, ** *p* < 0.01). NS indicates that the difference is not significant.

The TG detection analysis of primary chicken embryo liver cells revealed a significant increase in TG content in the SO/PA mixed treatment group compared to both the control group and the SO treatment group (Figure 6A). Furthermore, the Oil Red O staining results indicated that SO and the combination of SO/PA exhibited a stimulatory effect on steatosis in primary chicken liver cells, leading to the formation of larger lipid droplets (Figure 6C). To enhance the validation of SO/PA steatosis conditions in vivo, we conducted in ovo feeding experiments. The results of TG content analysis indicated a more pronounced effect of the SO/PA group in promoting fat deposition compared to the SO group (Figure 6B). Furthermore, Oil Red O staining of chicken embryo liver tissue demonstrated a deeper red color in the SO/PA group compared to the SO and NC group (Figure 6D). These findings suggested that the combination of SO/PA treatment successfully induced steatosis in primary chicken embryonic liver cells and embryonic liver.

**Figure 7:**
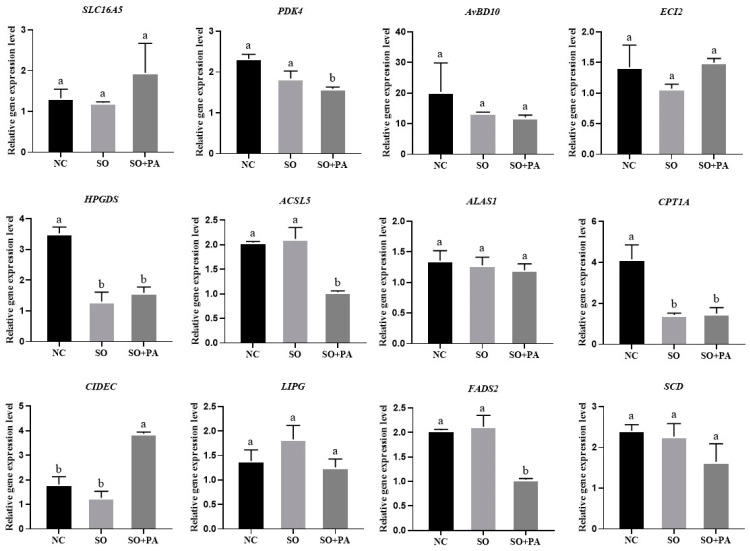
Gene expression levels of differentially expressed genes in primary chicken embryo liver cells under different induction conditions. Abbreviations: NC represents negative control; SO means 0.375 mM sodium oleate; SO + PA represents the mixture of sodium oleate and palmitic acid. Different lowercase letters indicate significant differences (*p* < 0.05).

**Figure 8:**
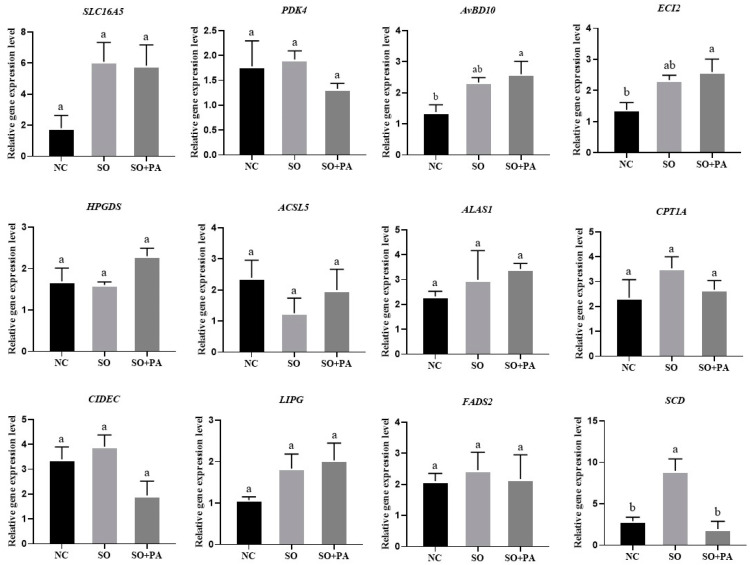
Gene expression levels of differentially expressed genes in chicken embryo liver tissue under different induction conditions. Abbreviations: NC represents negative control; SO means 0.375 mM sodium oleate; SO + PA represents the mixture of sodium oleate and palmitic acid. Different lowercase letters indicate significant differences (*p* < 0.05).

We also detected the gene expression levels of DEGs in primary chicken liver cells and embryonic liver tissue. The RT-qPCR results showed that the expression level of CIDEC in the SO/PA group was significantly higher than that in other groups in primary liver cells, while the expression levels of *PDK4*, *HPGDS*, *ACSL5*, *CPT1A*, and *FADS2* genes in the SO/PA group were significantly lower than those in the NC group. There was no significant difference in the expression levels of other genes among the groups (Figure 7). In the in ovo feeding experiment, *AvBD10* and *ECI2* were significantly higher in the SO/PA group than in the NC group, and the expression level of *SCD* was significantly higher in the SO group than in the other groups. The expression levels of other genes did not show significant differences among the groups (Figure 8).

## 4. Discussion

In recent years, liver lipid metabolism has been a research hotspot in humans, mice, and poultry [14,24,25,26]. Developing an optimal in vitro cell steatosis model is essential for enhancing comprehension of the metabolic pathways involved in steatosis and identifying potential metabolic regulators. While well-established models for cellular lipid accumulation exist in research involving humans and other mammals [13,16,17], limited literature exists on in vitro models of steatosis in poultry cells [19,20]. Consequently, this study systematically assessed the impact of various types and combinations of lipid solutions on the steatosis of chicken liver cells and utilized RNA-seq analysis to comprehensively investigate the molecular mechanisms involved.

Previous studies frequently employed OA and dexamethasone for a 24 h treatment of primary chicken liver cells to induce steatosis [9,27]. However, our research demonstrated that a 12 h OA treatment had limited effectiveness in inducing steatosis in both LMH cells and primary chicken liver cells. Therefore, we suggest that future investigations avoid utilizing OA for short-term induction of steatosis in poultry liver cells.

In this study, we have determined that SO exhibits the most stable steatosis induction effect at both low and high concentrations (Figure 1A). However, it is worth noting that while SO induces a significant increase in cellular TG content, it was not as pronounced as the increase induced by PA at equivalent concentrations. As a result, we employed a mixed solutions approach in our study to induce cellular steatosis, with the aim of achieving a more effective steatosis outcome. Our study revealed that the induction effect of the SO/PA solution was significantly superior to that of individual components SO and PA. This finding may be attributed to the heightened cytotoxicity of SP, leading to increased cell death and subsequently diminished steatosis. Additionally, PA exhibited greater lipid toxicity toward cells in comparison to OA [28,29]. Furthermore, our analysis of protein concentration indicated a lower level in the SP group as opposed to the PA group. The accumulation of TG has the potential to mitigate the lipid toxicity induced by fatty acids [30]. It is plausible that the increased generation of TG in the SO/PA group serves to alleviate the lipid toxicity associated with PA.

In the RNA-seq analysis, it was observed that the number of DEGs in the SO/PA group exceeded that of the SO treatment group (Figure 2D, Tables S4–S9), suggesting that the combined solution exerts a more pronounced impact on cellular gene expression. Furthermore, functional enrichment analysis revealed that the majority of pathways enriched in DEGs identified by the SO treatment group were closely associated with fatty acid metabolism. Conversely, in the mixed treatment group of SO/PA, a higher proportion of genes were found to be linked to programmed cell death GO terms. The mixture we have explored may have stronger lipid toxicity on cells, and therefore, its induction of cellular steatosis may differ from that of SO. The specific differences can be reflected in these DEGs (Tables S4–S9). For instance, the enzyme PDK4 exerts inhibitory control over the process of fatty acid biosynthesis and influences cellular respiration [31,32]. In the context of lipid solution treatments, PDK4 expression was notably reduced across all groups, potentially as a mechanism to suppress fatty acid synthesis. Additionally, the enzyme LIPG is involved in regulating TG metabolism [33], while heightened expression of HPGDS in adipocytes can lead to a marked decrease in inflammation and impact lipid metabolism [34,35]. Furthermore, the transporter SLC16A5 may serve a critical function in maintaining the balance of lipid and amino acid metabolism [36,37]. FADS2 [38], ACSL5 [39], SCD [40], and CPT1A [41] are all related to lipid metabolism. Moreover, AvBD10 has very strong antibacterial properties [42] and is highly expressed in the yolk sac of the embryo [43]. During the process of steatosis, it may activate the innate immunity of cells, which requires further experimental verification. Moreover, this study identified a large number of lipid metabolism-related genes through RNA-seq. With the help of the published poultry pan genome [44,45], various variations of the functional genes related to lipid metabolism will be discovered in the future.

Furthermore, significant enrichment of GO terms related to fatty acid metabolism, such as organic acid metabolic process, carboxylic acid metabolic process, and oxoacid metabolic process, as well as the KEGG pathway PPAR signaling pathway, fatty acid metabolism, and fatty acid biosynthesis, was observed in the NC vs. SO groups. While in the NC vs. SO + PA groups, enrichment of GO terms associated with cell death processes, including positive regulation of apoptotic process, positive regulation of programmed cell death, and positive regulation of cell death, along with the KEGG pathway taurine and hypotaurine metabolism, was significantly enriched. Additionally, in SO vs. SO + PA groups, the fatty acid metabolism processes were most significantly enriched. The analysis of protein–protein interaction networks revealed that core and hub genes in SO-treated groups were associated with fatty acid metabolism, including *SCD* [40] and *ACACB* [46], whereas in SO/PA-treated groups, they were related to cell proliferation, such as *CXCL12* [47] and *RGS1* [48]. Both functional enrichment analysis and protein–protein interaction network analysis of DEGs indicated that various lipid mixture treatments resulted in significant enrichment of distinct biological processes. This indicates that following the 12 h treatment period, alterations in the expression of key genes related to fatty acid metabolism were observed in the SO treatment group, but the SO/PA treatment group exhibited more TG production. The SO/PA-treated LMH cells showed signs of programmed cell death, such as apoptosis, potentially due to excessive fat accumulation or the toxicity of the combined solution. Of course, the RNA-seq results mainly reflect the disturbance of gene expression in LMH cells after steatosis. To further elucidate the molecular mechanism of lipid solution-induced cell steatosis, it is necessary to perform RNA-seq and proteomics at different time periods of induction.

The optimal conditions for steatosis identified on LMH cells were applied to primary chicken embryonic liver cells. Analysis of Oil Red O staining results revealed minimal variation among different groups. However, larger lipid droplets were observed in the SO/PA and SO groups compared to the NC group, suggesting that lipid solutions induce steatosis by increasing lipid droplet size. The formation and size of lipid droplets are of great significance for energy storage and overall bodily health [49,50], with the CIDE protein playing a pivotal role in their development [51,52]. *CIDEA* and *CIDEC* were significantly differentially expressed in the RNA-seq, and *CIDEC* was significantly highly expressed in the SO/PA group in chicken embryonic liver cells. It was reported that *CIDEA* and *CIDEC* may play important roles in regulating liver lipid metabolism in broilers [53]. In our future research, we will investigate the roles of *CIDEA* and *CIDEC* in the development of steatosis in avian liver cells. Additionally, primary liver cells had less severe steatosis effects compared to LMH cells possibly due to the absence of fetal bovine serum in cultivation. Future studies will explore longer cultivation times or alternative methods to induce steatosis.

We also applied the optimal conditions for steatosis in LMH cells to chicken embryonic liver tissue. During in ovo feeding experiments, we established various concentration gradients. However, we observed that excessively high concentrations, particularly of mixed SO/PA, rendered the acidic solution insoluble in our PBS injection, resulting in suboptimal steatosis effects. Conversely, under low concentration conditions, we noted a significantly greater induction effect of SO/PA on in vivo steatosis compared to the SO and NC group. This method can create a live fatty liver model at hatching for research purposes. However, RT-qPCR results showed inconsistent gene expression in primary cells and tissues with the LMH cells, possibly due to experimental variability. In the experiment, gender, genetics, and other factors should be considered, and this study did not conduct gender identification. Subsequent experiments will unify the factors of gender and egg weight, which may yield more consistent results.

Due to the similarity of primary fat formation in chicken liver to humans, chickens are an ideal animal model for understanding NAFLD [6]. This study demonstrated a novel approach to induce steatosis in LMH cells, primary chicken embryonic liver cells, and chicken embryonic liver tissue by utilizing a combination of SO and PA solution. The newly discovered method of inducing steatosis showed great efficacy to the 0.375 mM SO approach. Employing a cell model with enhanced steatosis induction capabilities to identify potential regulators or additives for improving lipid metabolism would strengthen the credibility of the findings. The research created a cellular model for screening effective additives for treating FLS or FLHS in poultry and exploring the underlying molecular mechanisms.

## 5. Conclusions

This study successfully induced steatosis in LMH cells through the utilization of various lipid solutions, ultimately determining that the optimal induction was achieved with the SO/PA (0.15/0.3 mM) solution over a 12 h period. Subsequent RNA-seq analysis revealed notable differences in the molecular mechanism of steatosis induced by SO/PA compared to SO alone, particularly in relation to genes linked to cell death. However, further validation is required to corroborate these findings. The optimal conditions for inducing steatosis in LMH cells were applied to primary chicken liver cells and chicken embryonic liver tissue through the in ovo feeding method. The outcomes of this experiment demonstrated a significant enhancement in steatosis following treatment with the SO/PA solution. Overall, this study has established an efficient method for inducing liver cell steatosis in chickens.

## Data Availability

The original data in this study are openly available in the NCBI SRA Database under accession number PRJNA1123141.

## Supplementary Materials

The following supporting information can be downloaded at https://www.mdpi.com/article/10.3390/ani14152173/s1, Table S1: Detailed primers information of RT-qPCR; Table S2: Summary of sample RNA-seq data quality; Table S3: The total reads and mapping results of RNA-seq data; Tables S4~S9: Detailed information of differentially expressed genes selected by RNA-seq data between different groups.
